# Clinical Values and Markers of Radiation-Induced Liver Disease for Hepatocellular Carcinoma With Portal Vein Tumor Thrombus Treated With Stereotactic Body Radiotherapy

**DOI:** 10.3389/fonc.2021.760090

**Published:** 2021-12-14

**Authors:** Jun Jia, Jing Sun, Xuezhang Duan, Wengang Li

**Affiliations:** Radiation Oncology Department, The Fifth Medical Center of Chinese People’s Liberation Army (PLA) General Hospital, Beijing, China

**Keywords:** HCC, SBRT, PVTT, RILD, radiation-induced liver disease

## Abstract

**Background:**

Information about radiation-induced liver disease (RILD) in hepatocellular carcinoma (HCC) patients preexisting hepatitis B cirrhosis with portal vein tumor thrombus (PVTT) extended to the main portal vein treated with stereotactic body radiotherapy (SBRT) is still inadequate and the predictive markers for RILD have not been cleared in these patients. The aim of the study is to identify factors that can be used to predict RILD and to evaluate the influence of RILD in these patients.

**Methods:**

In our study, 59 patients were analyzed and evaluated from December 2015 to June 2019, according to the entry criteria. After treatment, 59 patients were followed up within the first month and then every 3 months. Hematology test, tumor markers, three-phasic CT scan of the lungs, and CT or MRI scan of the liver were performed at each follow up.

**Results:**

Median overall survival time was 10.7 months (range, 5.8 to 14.9). RILD appeared in 17 of the 59 patients (28.8%) at the 3rd month after SBRT. In the univariate analysis, not only the CP score class (A or B) but also each different pretreatment CP score (*p* < 0.05) was a significant predictive factor of RILD. More RILD cases were detected with the increase of CP score. The recovery rate decreased as the baseline CP score increased (*p* < 0.05). It was found that the overall survival time was affected by only baseline CP score and RILD (*p* < 0.05).

**Conclusions:**

The development of RILD has a dependency on the CP score in these patients. CP scores before treatment and RILD are significantly associated with overall survival. SBRT is an effective and safe method for patients with CP ≤ B7. For patients with CP-B8, liver function should be monitored more frequently. It is not safe enough for the SBRT treatment in CP-B9 patients.

## Introduction

Hepatocellular carcinoma (HCC) is a kind of tumor with very high malignancy. The incidence of HCC ranked 6th among all types of cancer worldwide. It has ranked third in the causes of cancer-related death in China up to now (1, 2). The first choice of treatment for small HCC is surgical resection. However, when being diagnosed many patients are unsuitable or hard for resection or other local treatments, especially the cases with macrovascular invasion (MVI) (3, 4). The most common form of MVI in HCC is portal vein tumor thrombus (PVTT), patients with which have a very short survival time, with an incidence ranging from 44 to 62.2%, and the prognosis for patients with PVTT remains poor till now (5). The treatment of HCC with extensive portal vein involvement remains disputed and intricate. In previous studies, patients with PVTT presented in the main or contralateral branch of portal vein had no survival benefits from surgical resection, whose survival times were generally even shorter (6). Treatments of HCC with PVTT extended to the main portal vein are multidisciplinary and include surgery, intervention such as trans-arterial chemoembolization (TACE), radiotherapy, tyrosine kinase inhibitors (TKIs), and programmed cell death protein 1 inhibitors (PD-1) (7, 8).

It can be learned from previous studies that in the cases of PVTT extended to the main portal vein, radiotherapy (RT) was often considered as a secondary treatment that might be effective (9, 10). Stereotactic body radiotherapy (SBRT), as an emerging technology, delivers high doses of radiation to the target in a few fractions, coupled with a high degree of accuracy in target delineation, which takes advantage of the advancements in precise radiation dose delivery, respiratory motion management, and precise image guidance (11–13). Nowadays, for the small HCC patients who are not qualified for surgery or other local treatments, SBRT has already been adopted as an effective treatment (14). By now, few studies have explored the efficacy and risk of SBRT for the treatment of PVTT involving the main trunk. Among the treatments of HCC patients in our center, SBRT is adopted for the patients who have unresectable HCC with PVTT now.

Meanwhile, side effects must be considered and managed in the use of SBRT. Because of limited effective treatments to cure various kinds of side effects, radiation-induced liver disease (RILD) is so important in the use of SBRT that more attention should be drawn to it. So, determining predictive factors for RILD seems important in clinical work. For some kinds of tumors, a few studies have found that dose-volumetric parameters were predictive factors of RILD (12, 15, 16). In addition, some clinical factors were related to the occurrence and development of RILD have been reported (13, 15–17). But the research is even less about RILD in patients with HCC, especially patients with PVTT extended to the main portal vein who were treated with SBRT. Many factors may increase the risk of RILD. In China, the main cause of liver cancer is hepatitis B cirrhosis. In clinical work, the great majority of patients with HCC in China have been preexisting hepatitis B, which also affects the occurrence and development of RILD (12). Information about RILD in HCC patients preexisting hepatitis B cirrhosis with PVTT presented in the main portal vein treated with SBRT remains inadequate.

The purpose of the study is to investigate suitable markers that affect RILD in patients who have been preexisting hepatitis B cirrhosis with PVTT presented in the main portal vein who are treated with SBRT and we followed the methods of Kim et al., 2018 (18).

## Data and Methods

### Ethics Statement

This study was approved by the Institutional Review Board of The Fifth Medical Center of PLA General Hospital and was conducted in accordance with the Declaration of Helsinki and internationally accepted ethical guidelines. All patients signed written informed consent for their information to be stored in the hospital databases and used for research.

### Clinical Data

From December 2015 to June 2019, 59 HCC patients with PVTT treated by SBRT in the Fifth Medical Center of PLA General Hospital were finally enrolled. The inclusion criteria were as follows: primary HCC with hepatitis B cirrhosis, pretreatment Child-Pugh (CP) class A or B with good performance status (0 or 1 score), HCC with PVTT presented in the main portal vein at the time of diagnosis, no evidence of extrahepatic metastasis, the follow-up studies ≥ 3 months, no other treatments were performed before SBRT, no other local treatment was taken within 3 months after SBRT, more than 700 cc of uninvolved liver.

### Therapeutic Method

Target tracking was provided by 4 to 6 golden fiducials implanted before receiving SBRT treatment (CyberKnife, Accuray, USA). Dynamic respiration tracking and fiducial tracking were applied simultaneously in the treatment. CT localization: A plain CT scan was done one week after implantation. Except for benchmark images, the additional images were chosen based on patients’ conditions, and these images were enhanced CT scans, magnetic resonance imaging (MRI), positron emission tomography-computed tomography (PET-CT), or hepatic arteriography, etc. SBRT treatment plan design: The gross tumor volume (GTV) represented the tumor thrombosis visualized on the contrast-enhanced CT or MRI. The gross tumor volume (GTV) and organs at risk (normal liver, kidneys, esophagus, stomach, duodenum, bowel, and spinal cord) were contoured by an oncologist and the planning target volume (PTV) was expanded 3–5 mm around the GTV, which contoured 100% of GTV (14). All plans were designed by G4 CyberKnife MultiPlan (Version 4.0.2). A total dose of 49-54Gy was delivered in 7–9 fractions (49Gy/7f, 48Gy/8f, or 54Gy/9f). The normal tissue dose was within the normal radiotherapy tolerance dose (TG-101).

### Follow-Up

Liver function assessment and routine blood examination were all done before treatment. After SBRT, the patients were followed up and liver function was examined within 1 month and then every 3 months until the death or necessary.

### Evaluation of RILD

The liver toxicity reaction evaluation is based on the definition of RILD, of which there are two types: classic RILD and non-classic RILD. Non-classic RILD occurs in patients with underlying chronic hepatic diseases. So, we refer to the non-classic RILD definition which is divided into elevated liver transaminases increased by more than fivefold compared to normal levels, or worsening of Child-Pugh (CP) score by 2 or more within 3 months after SBRT. Meanwhile, a decrease of CP score or normalization of hepatic enzymes within 6 months could be defined as recovery from RILD (19). CP score was recorded at the beginning of SBRT within 1 week. The clinical data analyzed were age, gender, CP score, alpha fetoprotein (AFP), aspartate aminotransferase (AST), and alanine aminotransferase (ALT). Dose-volumetric data analyzed were SBRT dose and dose per fraction. Liver function, coagulation function, and routine blood tests were examined regularly during the SBRT procedure to evaluate acute toxicity every 3-5 days. After treatment, all patients were followed up within 1 month, and then every 3 months.

### Statistical Methods

SPSS 26.0 software was used to perform statistical analyses. Differences between groups were compared according to the Chi-square test, or Student *t* test. The overall survival (OS) was calculated from the diagnosis to the date of either death or the last follow-up visit. Cox regression analysis was used to predict the effective markers for survival time. P-values less than 0.05 were considered to indicate statistically significant differences.

## Results

### Patient Characteristics

Baseline characteristics of the patients are summarized in **Table 1**. The follow-up time of the patients is 14.9 months. According to the admission criteria for this study, 59 patients were analyzed and evaluated. Overall survival time was defined as the time from diagnosis to death. The median overall survival time was 10.7 months (range, 5.8 to 14.9). The median age was 57 years old (range, 33 to 74). RILD was observed in 17 of the 59 patients (28.8%) in the 3rd month after the end of SBRT. An increase of CP score by 2 appeared in 13 of the 17 patients (76.5%). A liver transaminases increase by more than fivefold compared to normal levels appeared in 4 of the 17 patients (23.5%). By definition, CP Class A includes CP scores 5 and 6, and CP Class B includes CP scores 7, 8, and 9. Of all the patients, 27 were in CP Class A and 32 were in Class B. The number of initial AFP increased (> 10 ng/ml) was 53, the initial ALT increase (> 35 U/ml) was 31 and the initial AST increase (> 40 U/ml) was 26. A total of 48 patients (81.4%) received tyrosine kinase inhibitors (TKIs) (23 Sorafenib, 25 Lenvatinib) after SBRT. There were 2 (4.7%) patients that received programmed cell death protein 1 inhibitors (PD-1) alone after SBRT, 5 patients (11.6%) that received SBRT alone,7 (11.9%) patients that received both PD-1 inhibitors and TKIs after SBRT, and 23 (38.9%) patients that received TACE when progression happened after SBRT.

**Table 1:** Baseline characteristics of the patients.

Characteristics	No. of patients (n = 59)
Age (years)	
Median	57
Range	33-74
Sex	
Male	45
Female	14
RILD	
yes	17
no	42
Child-Pugh class	
A	27
B	32
Child-Pugh score	
5	14
6	13
7	16
8	14
9	2
Radiation dose (Gy)	
Median	48
Range	45-49
Fraction	8
Median	7-9
Range	
Treatment after SBRT	
TKIs alone	41
PD-1 alone	2
TKIs and PD-1	7
TACE	23
Baseline marker levels	
AFP>10ng/ml	53
ALT>35U/L	31
AST>40U/L	26
Overall Survival	10.7
Median	5.8-14.9
Range	

### Markers of RILD

In the univariate analysis, not only CP score class (A or B) but also different pretreatment CP score (*p* < 0.05) is the significant predictive factor of RILD. By analyzing, it is found that age, gender, baseline AST, baseline ALT, baseline AFP, SBRT dose, and dose per fraction do not have an obvious correlation to RILD (**Table 2**). Different scores are found to be significantly associated with RILD (*p* < 0.05). From the data analyzed separately, compared with CP-A5 patients, the incidence of RILD in CP-A6 patients and CP-B7 patients does not increase statistically, while the increase of the incidence of RILD in CP-B8 and CP-B9 patients has statistical significance. For the incidence of RILD, the CP-B7 patients may be a watershed in the treatment of the patients who have been preexisting hepatitis B cirrhosis with PVTT extended to the main portal vein [CP score 6 (CP-A6): 95% confidence interval (CI), -0.407–0.243; *p*=0.613] [CP score 7 (CP-B7): 95% CI, -0.550–0.068; *p*=0.123] [CP score 8 (CP-B8): 95% CI, -0.747–0.110; *p*=0.009] [CP score 9(CP-B9): 95% CI, -1.566–0.291; *p*=0.005] (**Table 3**).

**Table 2:** Patient characteristics in relation to the risk of RILD.

Variable	RILD (n=17)	Non-RILD (n=42)	p value
Sex			0.518
Male	12	33	
Female	5	9	
Age (year)	57 (42-69)	57 (33-73)	0.881
CP score			0.047
5	1	13	
6	2	11	
7	5	11	
8	7	7	
9	2	0	
CP score class			0.009
A	3	24	
B	14	18	
Radiation dose (Gy)			
Median	48	49	0.102
Range	45-49	45-49	
Fraction			
Median	8	8	0.197
Range	7-9	7-9	
Baseline markers			
AFP	43 (128 ± 49.991)	14 (40 ± 10.680)	0.179
ALT	45 (51.176 ± 5.757)	32 (45.615 ± 6.074)	0.919
AST	54 (48.471 ± 3.988)	54.5 (40.154 ± 6.270)	0.584
Overall survival			
Median	7.8	9.4	≤0.001
Range	4.7-9.2	4.7-12.5	
Recovery			
Yes	6		
No	11		

**Table 3:** Each different score in relation to the risk of RILD.

Variable	95%CI	*p* value
Child-Pugh score		
5	–	–
6	-0.407–0.243	0.613
7	-0.550–0.068	0.123
8	-0.747–0.110	0.009
9	-1.566–0.291	0.005

### RILD and CP Score

**Figure 1:**
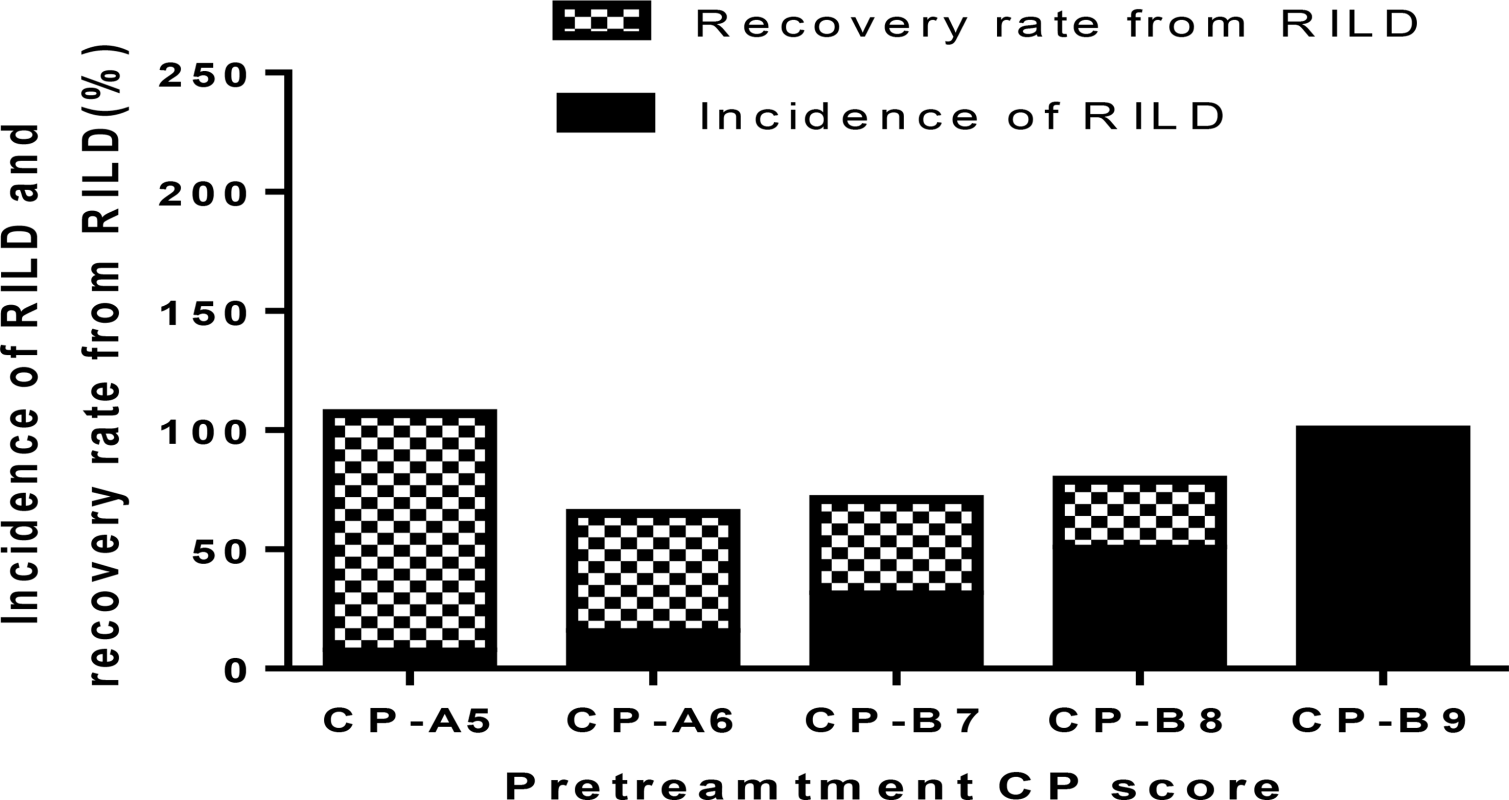
The incidence of RILD and recovery rate after SBRT in different CP scores.

The occurrence of RILD is correlated with the baseline CP score, especially the CP > B7 patients. The incidence of RILD after SBRT is 7.14% (1 of 14 patients) in CP-A5 patients, 15.38% (2 of 13 patients) in CP-A6 patients, 31.25% (5 of 16 patients) in CP-B7 patients, 50.00% (7 of 14 patients) in CP-B8 patients, and 100.00% (2 of 2 patients) in CP-B9 patients. The incidence of RILD increases clearly above CP-A6, and it has statistical significance above CP-B7. The recovery rate decreases as baseline CP score increases. The recovery rate is 100.00% (1 of 1 patients) in CP-A5 patients, 50.00% (1 of 2 patients) in CP-A6 patients, 40.00% (2 of 5 patients) in CP-B7 patients, 28.57% (2 of 7 patients) in CP-B8 patients, and 0.00% (0 of 2 patients) in CP-B9 patients. The recovery rate decreases evidently in patients above CP-B7 (**Figure 1** and **Table 4**).

**Table 4:** Incidence and recovery rate.

	Incidence rate of RILD (%)	Recovery rate from RILD (%)
CP-A5	7.14	100.00
CP-A6	15.38	50.00
CP-B7	31.25	40.00
CP-B8	50.00	28.57
CP-B9	100.00	0.00

### Changes of Liver Function

**Figure 2:**
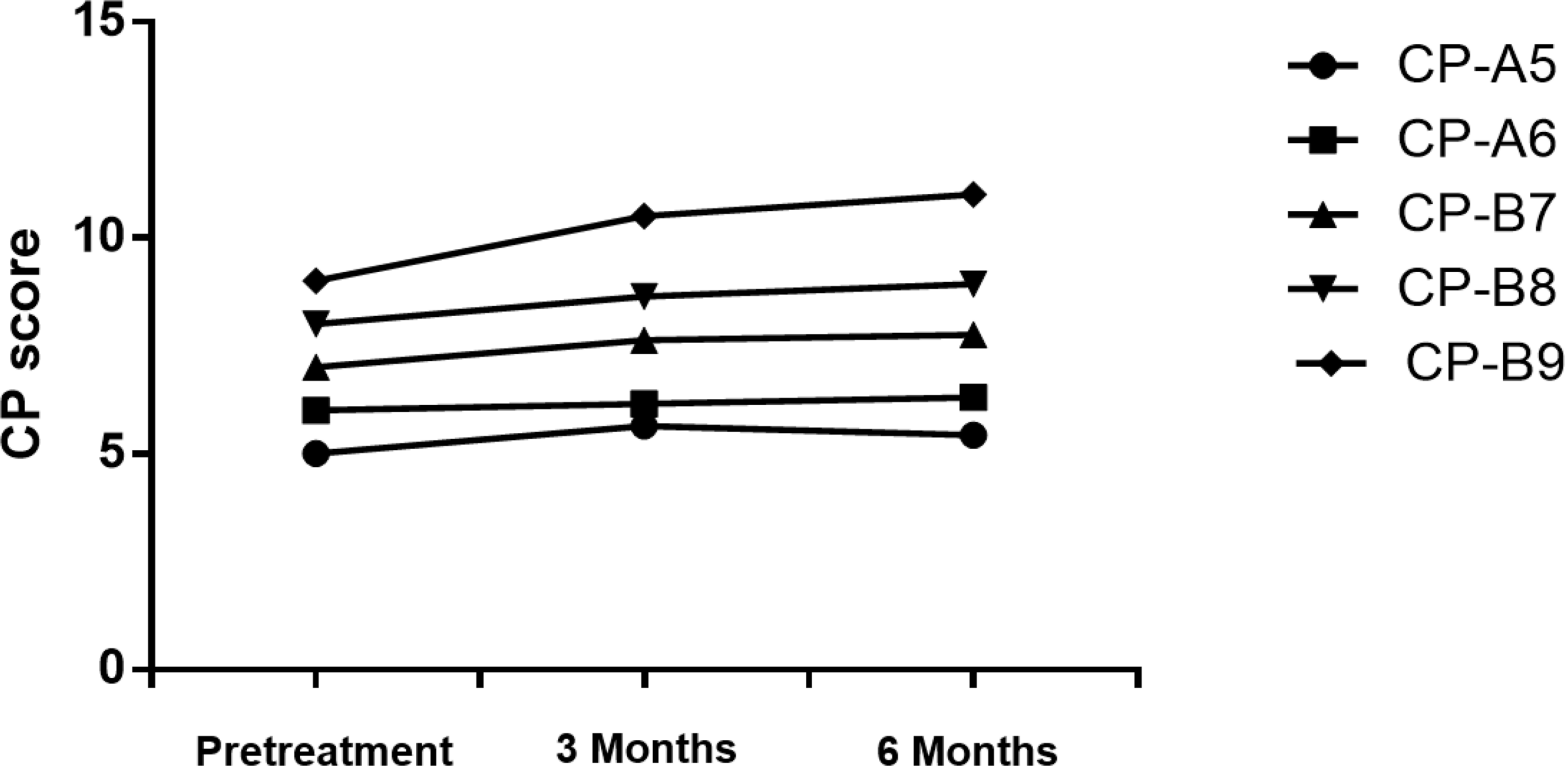
The change of CP score at the 3rd and 6th month.

We examined patients’ liver function and calculated CP scores at the 3^rd^ and 6th months after SBRT (**Figure 2** and **Table 5**). With the increase of CP score stage, the variance of CP score after SBRT became larger. The change in CP score is 0.64, 0.15, 0.63, 0.64, and 1.5 points in patients with CP-A5, CP-A6, CP-B7, CP-B8, and CP-B9 at the 3rd month, respectively. The change in CP score is -0.21, 0.16, 0.12, 0.29, and 0.5 points in patients with CP-A5, CP-A6, CP-B7, CP-B8, and CP-B9 at the 6th month compared to those at the 3rd month, respectively. We can see that in the 6th month, with the increase of baseline CP score, the change range of CP score also increases. There was 1 patient with CP-B9 who died within 6 months because of hepatic failure.

**Table 5:** Variance of CP score.

	Pretreatment	3 Months	6 Months
CP-A5	5	5.64	5.43
CP-A6	6	6.15	6.31
CP-B7	7	7.63	7.75
CP-B8	8	8.64	8.93
CP-B9	9	10.5	11

### Investigation of Survival Time

**Figure 3:**
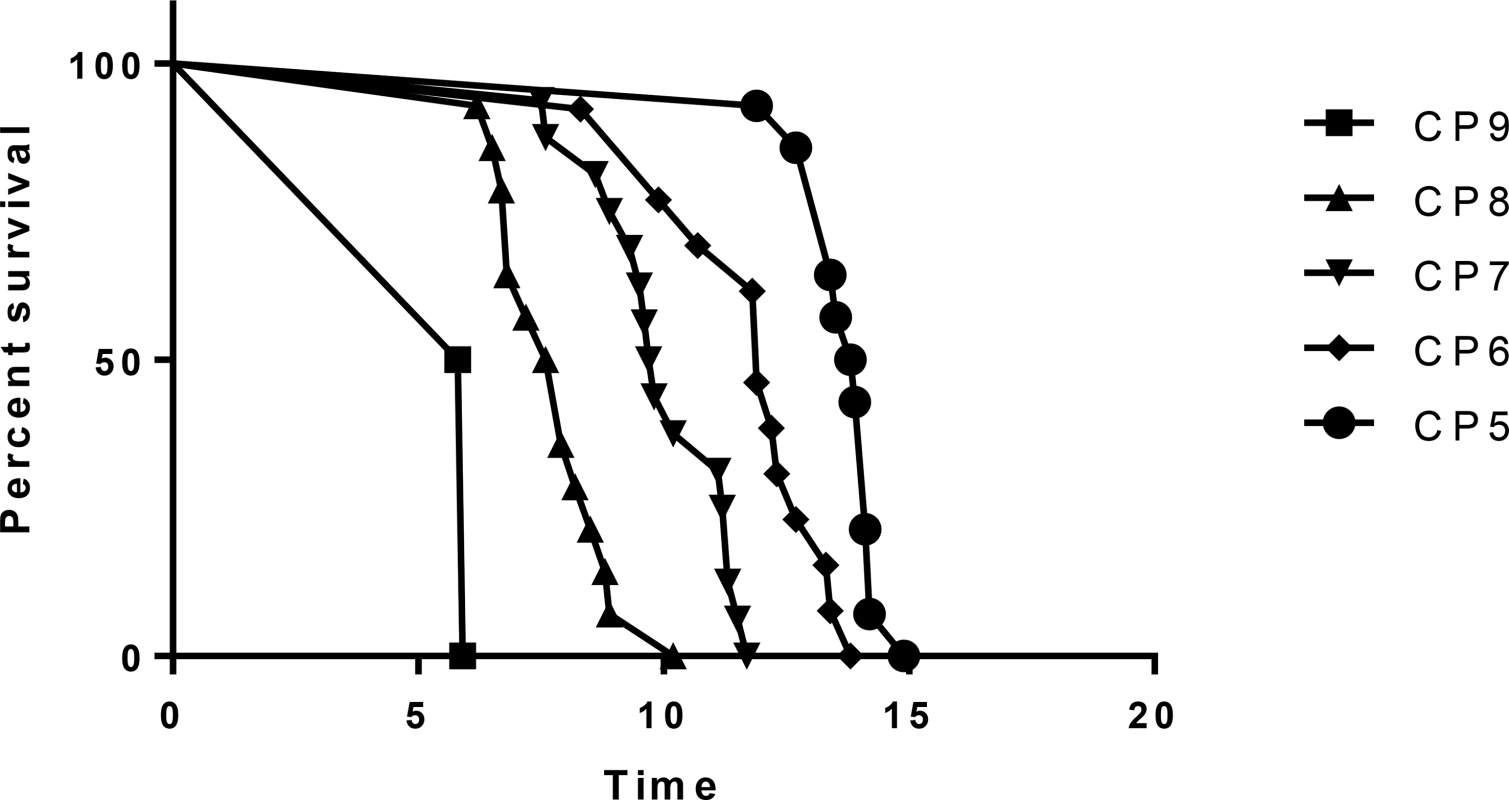
Overall survival in different CP scores.

In the Cox regression analysis, the overall survival time is affected by baseline CP score and RILD. The relationship between age, baseline ALT/AST/AFP, and survival time is not statistically significant (p>0.05) (**Table 6**). For these patients treated with SBRT, with the increase of baseline CP score, the survival time decreases significantly. And for the patients with similar basic characteristics, the occurrence of RILD affects the survival time. This means that once RILD occurs, survival may be impaired. For patients with low CP scores and without RILD, the survival time is longer (CP-A6: hazard ratio (HR), 0.003; 95% CI, 0.000 to 0.107; *p* = 0.001) (CP-B7: HR, 0.009; 95% CI, 0.000 to 0.208; *p* = 0.003) (CP-B8: HR, 0.023; 95% CI, 0.001 to 0.372; *p* = 0.008) (CP-B9: HR, 0.094; 95% CI, 0.004 to 0.645; *p* = 0.022) (RILD: HR, 1.007; 95% CI, 0.347 to 2.880; *p* = 0.04) (**Figure 3** and **Table 6**). Of the 17 RILD patients, 1 (5.88%) died of RILD within 6 months after SBRT. Death related to RILD occurred only in patients with CP-B9.

**Table 6:** Analysis of overall survival.

Variable	HR	95%CI	p value
Age	1.011	0.951-1.075	0.726
AST	1.006	0.987-1.025	0.568
ALT	0.998	0.979-1.018	0.851
AFP	1.0002	0.999-1.004	0.155
Child-Pugh score			0.024
5			
6	0.003	0.000-0.107	0.001
7	0.009	0.000-0.208	0.003
8	0.023	0.001-0.372	0.008
9	0.049	0.004-0.645	0.022
RILD	1.007	0.347-2.880	0.04

### Possible Effects of Follow-Up Treatment

Overall survival time is 10.93 months for those who received TKIs after SBRT, 9.78 months for those receiving PD-1 inhibitors after SBRT, and 11.94 months for those who received TACE after SBRT when progression happened. Although the overall survival time is not statistically significant in this study, we can still find some trends. The use of systemic therapy may help improve survival under the premise of fully considering the possible toxic reactions.

## Discussion

For HCC patients, in addition to the malignant degree of the tumor, the survival time is also affected by the degree of liver cirrhosis. The more severe the liver cirrhosis is, the worse the liver function is, and the worse tolerance to various anti-tumor treatment methods is. In China, the main cause of liver cirrhosis is hepatitis B cirrhosis. In addition, for patients with PVTT, the pressure of the portal vein increases, and the incidence of liver failure and gastrointestinal bleeding increases significantly. Previous studies have shown that when the cancer thrombus existed in the main portal vein, the median survival time was only 5.6 months, especially in patients with liver function grade B, whose median survival time was less than 3 months (20). It can be concluded from the above that if there is a main portal vein tumor thrombus in hepatitis B related HCC patients, there is a great challenge in the anti-tumor treatment, as such patients have short survival periods, limited treatment options, and a high risk of hepatic failure. Therefore, we consider that for such patients, the implementation of SBRT for portal vein tumor thrombus may open up a new situation. In present studies, the patients with PVTT, especially involving the main trunk, were unsuitable for surgery or TACE due to the failure in prolonging the survival time, while traditional radiotherapy might improve the risk of hepatic failure. In recent years, SBRT has emerged to be a critical treatment for such patients. But the risk of RILD for SBRT is still uncertain for those patients. So, markers to predict RILD need to be established to help us to avoid the occurrence of RILD as far as possible. In some literatures, RILD is divided into “classic” and “non-classic” types (19). Classic RILD typically occurs at about 1 month after the completion of radiotherapy. Some previous studies showed that non-classic RILD was discovered in patients with underlying chronic liver disease, so RILD is defined as the non-classic in our study (15, 21, 22). In our study, RILD was found in 17 of the enrolled 59 patients. The results showed that CP score before treatment was significantly associated with the occurrence of RILD. Compared with CP-A5 patients, the incidence of RILD in CP-A6 patients and CP-B7 patients did not increase statistically, while the increase of the incidence of RILD in CP-B8 and CP-B9 patients had statistical significance. Previously, some studies also reported that ALT and AST were important factors to predict RILD (17, 23), but we did not find the relevance in our study. According to Kimura T et al. (23), the incidence rate of RILD was higher in the CP class B than class. A. Jung J et al. (17) have found that the marker related to grade 3 liver toxicity or greater was CP score. We followed the methods of Kim et al. to find more information about RILD in HCC patients preexisting hepatitis B cirrhosis with PVTT extended to the main portal vein treated with SBRT (18). In our study, we chose a group of patients who were prone to radiation injury, as all the patients enrolled had hepatitis B cirrhosis. The patients with different CP scores do not have the same liver function in clinical work. We analyzed the relation of the incidence rate of RILD and CP score. The probability of RILD is related to the stage of CP score (*p* < 0.05) (**Table 3**). This suggests that for patients in our study, especially in cases of CP class B, there may be differences in the development of RILD. For the incidence of RILD, the CP-B7 patients may be a watershed in the treatment of these patients. Some studies have reported that the liver function of patients with CP-A6 may be inferior to that of CP-A5 patients because of fibrosis (24, 25). So, there are reasons to believe that CP > B7 patients have a higher probability of RILD than others. Patients with different CP scores have different risk probabilities. There may be differences between CP-A5, CP-A6, and CP-B7, but perhaps due to the number of cases, there is no statistical difference in our study.

The recovery rate from RILD is examined and the dynamic changes of CP score after SBRT are described. The recovery rate is 100% in CP-A5 patients. It is moderate in CP-A6 (50%) and CP-B7 (40%) patients. The recovery rate is clearly lower in CP ≥ B8 patients: 28.57% in CP-B8 patients and 0% in CP-B9 patients (**Figure 1** and **Table 4**). That RILD is not safe enough in CP-B9 patients can be concluded due to the high possibility of RILD and low recovery rate from it. For the CP-B8 patients, treatment should be evaluated carefully because the risk of RILD is high and liver function should be assessed more frequently because the recovery rate is clearly lower than the CP < B8 patients. Several other studies also supported that it was not safe enough in CP ≥ B8 patients during the treatment of SBRT. Nabavizadeh N. suggested that only CP ≤ B7 patients may be suitable for SBRT because of the moderate liver toxicity (26). Another study has reported that CP ≥ B8 is associated with serious liver toxicity or death (*p* = 0.030) (23). We can conclude that in our study, for the patients with PVTT extended to the main portal vein, more attention should be given during and after the treatment of SBRT in CP ≥ B8 patients.

It is obvious that SBRT could affect liver function. However, few studies about it have been done. Wo JY et al. have reported that decline in CP classification in HCC patients is the most important and common change after SBRT (27). In this study, we evaluate CP score and liver function at the 3rd and 6th months after SBRT. In patients with CP-A5, CP-B7, and CP-B8, the CP score changes by 0.5-1 point at the 3rd month. However, different from the other two groups, the scores of CP-A5 patients dropped at the 6th month. So, we find that the change in function of the liver is more clear in CP ≥ B7 patients than it in CP < B7 patients. But for the CP-B9 patients, the CP score changes by 1.5 points at the 3rd month. The CP class evolves from class B to C (**Figure 2** and **Table 5**). The results show that the CP-A5 and CP-A6 patients with PVTT extended to the main portal vein are safe, in contrast, as they can tolerate the liver toxicity caused by SBRT. In the patients with CP-B7, it is neccessary to monitor liver function carefully to avoid RILD as far as possible. In patients with CP-B8, more methods should be tried to adjust dose per fraction and total dose and monitor liver function more frequently. In patients with CP-B9, it is not safe enough for the SBRT treatment.

The results show that CP score before treatment and RILD are significantly associated with overall survival. Previously, Xu ZY et al. (16) and Son SH et al. (28) have shown that the overall survival time could be shorted by RILD. It can be concluded in our study, the overall survival is also related to RILD statistically for the hepatitis B patients with PVTT extended to the main portal vein. Especially in the patients with poor baseline liver function (CP ≥ B8, especially B9), RILD seems an important factor that can influence overall survival time. The correlation between initial CP score and survival time is obvious too. Patients with different scores have different survival times. It was reported by Kimura T et al. that CP ≥ B8 patients were more probably subjected to severe liver toxicity and the death rate was rising (23). In some other research, the CP-B7 patients had a longer survival time and less toxicity caused by radiotherapy than CP ≥ B8 patients (29). In the light of these studies, CP ≥ B8 patients might have irreversible damage of liver function, thus the survival time could be shorted. Therefore, once RILD occurs, it is hard for these patients to reverse the damage. For these patients treated with SBRT, with the increase of baseline CP score, the survival time decreases significantly. And for the patients with similar basic characteristics, the occurrence of RILD affects the survival time. The survival time of patients with poor liver function is shorter as we know. While the occurrence of RILD means that these patients have poor tolerance to SBRT. So, it is not hard to understand the impact of these two factors on survival time.

Our study also has some limitations. First, it is a retrospective study. Second, the number of cases is relatively small, because we include only HCC patients who have been preexisting hepatitis B cirrhosis with PVTT extended to the main portal vein treated by SBRT. More CP-B Class patients need to be treated with SBRT to find more markers to reduce the incidence of RILD for clinicians. Further prospective investigation is needed to clarify more relationships between liver function, RILD, and survival time. Due to the small number of cases and the lack of previous experience, the combined therapy of TKIs, PD-1 inhibitors, and SBRT failed to show a clear survival advantage, statistically. If there is no contraindication, TACE is still a better choice for patients with tumor progression after SBRT. After we have accumulated the experience of treatment, multidisciplinary treatment needs to be proven to be the direction of the future in more prospective investigations.

## Conclusion

CP score is closely related to the development of RILD in patients who have been preexisting hepatitis B cirrhosis with PVTT extended to the main portal vein. CP score before treatment and RILD are significantly associated with overall survival. SBRT is a safe and effective treatment for patients with CP ≤ B7. For patients with CP-B8, more methods should be tried to adjust dose per fraction and total dose and monitor liver function more frequently. It is not safe enough for the SBRT treatment in CP-B9 patients. The use of systemic therapy may help improve survival under the premise of fully considering the possible toxic reactions.

## Data Availability Statement

The original contributions presented in the study are included in the article/s **Supplementary Material**. Further inquiries can be directed to the corresponding author.

## Ethics Statement

This study was approved by the Institutional Review Board of The Fifth Medical Center of PLA General Hospital and was conducted in accordance with the Declaration of Helsinki and internationally accepted ethical guidelines. All patients signed written informed consent for their information to be stored in the hospital databases and used for research.

## Author Contributions

Data analysis and interpretation, and drafting and revision of the manuscript for critically important intellectual content JJ, data acquisition JS, manuscript preparation JJ, and provision of final approval of the version to be published WL and XD. All authors have read and approved the final version.

## Funding

This study protocol was supported by a grant from the National Natural Science Foundation of China 81972856.

## Conflict of Interest

The authors declare that the research was conducted in the absence of any commercial or financial relationships that could be construed as a potential conflict of interest.

## Publisher’s Note

All claims expressed in this article are solely those of the authors and do not necessarily represent those of their affiliated organizations, or those of the publisher, the editors and the reviewers. Any product that may be evaluated in this article, or claim that may be made by its manufacturer, is not guaranteed or endorsed by the publisher.
